# Prevalence and Determinants of Medication Adherence among Patients with HIV/AIDS in Southern Vietnam

**DOI:** 10.3390/idr13010014

**Published:** 2021-02-05

**Authors:** Phuong M. Nguyen, Anh N. Thach, Xuan D. Pham, Anh N. Lam, Thao N. P. Nguyen, Chu X. Duong, Lam V. Nguyen, Thao H. Nguyen, Suol T. Pham, Katja Taxis, Thang Nguyen

**Affiliations:** 1 Department of Pediatrics, Can Tho University of Medicine and Pharmacy, Can Tho 900000, Vietnam; nmphuong@ctump.edu.vn; 2 Soc Trang Center for Disease Control, Soc Trang 96000, Vietnam; anhpacst@gmail.com; 3 Department of Biomedical Sciences and Engineering, National Central University, Taoyuan 320317, Taiwan; pdinhxuan@gmail.com; 4 Department of Epidemiology, Can Tho University of Medicine and Pharmacy, Can Tho 900000, Vietnam; lnanh@ctump.edu.vn; 5 Department of Pharmacology and Clinical Pharmacy, Can Tho University of Medicine and Pharmacy, Can Tho 900000, Vietnam; nnpt1997@gmail.com (T.N.P.N.); dxchu@ctump.edu.vn (C.X.D.); ptsuol@ctump.edu.vn (S.T.P.); 6 Department of Anatomy, Can Tho University of Medicine and Pharmacy, Can Tho 900000, Vietnam; nvlam@ctump.edu.vn; 7 Department of Clinical Pharmacy, University of Medicine and Pharmacy at Ho Chi Minh City, Ho Chi Minh 710000, Vietnam; huongthao0508@gmail.com; 8 Groningen Research Institute of Pharmacy, University of Groningen, 9713 AV Groningen, The Netherlands; k.taxis@rug.nl

**Keywords:** human immunodeficiency virus (HIV), acquired immune deficiency syndrome (AIDS), antiretroviral therapy (ART), medication adherence, Vietnam

## Abstract

This study was conducted to determine the prevalence and determinants of medication adherence among patients with HIV/AIDS in southern Vietnam. Methods: A cross-sectional study was conducted in a hospital in southern Vietnam from June to December 2019 on patients who began antiretroviral therapy (ART) for at least 6 months. Using a designed questionnaire, patients were considered adherent if they took correct medicines with right doses, on time and properly with food and beverage and had follow-up visits as scheduled. Multivariable logistic regression was used to identify determinants of adherence. Key findings: A total of 350 patients (from 861 medical records) were eligible for the study. The majority of patients were male (62.9%), and the dominant age group (≥35 years old) accounted for 53.7% of patients. Sexual intercourse was the primary route of transmission of HIV (95.1%). The proportions of participants who took the correct medicine and at a proper dose were 98.3% and 86.3%, respectively. In total, 94.9% of participants took medicine appropriately in combination with food and beverage, and 75.7% of participants were strictly adherent to ART. The factors marital status (odds ratio (OR) = 2.54; 95%CI = 1.51–4.28), being away from home (OR = 1.7; 95%CI = 1.03–2.78), substance abuse (OR = 2.7; 95%CI = 1.44–5.05), general knowledge about ART (OR = 2.75; 95%CI = 1.67–4.53), stopping medication after improvement (OR = 4.16; 95%CI = 2.29–7.56) and self-assessment of therapy adherence (OR = 9.83; 95%CI = 5.44–17.77) were significantly associated with patients’ adherence. Conclusions: Three-quarters of patients were adherent to ART. Researchers should consider these determinants of adherence in developing interventions in further studies.

## 1. Introduction

The global human immunodeficiency virus (HIV) epidemic is one of the major worldwide health issues. In recent years, thanks to a myriad of efforts, the pandemic has been alleviated; there were 690,000 acquired immune deficiency syndrome (AIDS)-related deaths at the end of 2019, down from a peak of 2.2 million in the mid-2000s [1,2]. Of note, the estimated 1.7 million HIV patients worldwide in 2019 marked a 23% decline in new infections since 2010 [2].

In Vietnam, new HIV infections significantly reduced from 16,000 cases in 2010 to 5200 cases in 2019 [2]. The decrease of 67.5% in new infections and 40% in AIDS-related deaths over a 10-year period speaks to the effectiveness of the nationwide testing and treatment program. Additionally, the 2019 HIV/AIDS prevention and control report highlighted that nearly 95% had an HIV load below the detection threshold (<200 copies/mL) in the first 9 months of 2019 [3].

In addition to the effectiveness of antiretroviral therapy (ART), patients’ adherence to medical treatment plays a critical role in those positive health outcomes. Prior research has emphasized the significant correlation of optimal ART adherence with being virally suppressed [4,5,6]. Hence, the assessment of adherence to ART and evaluation of maintenance-associated factors in patients are critical.

There are few studies figuring out the correlation between patients’ characteristics and their medical adherence as well as the current status of ART adherence at a provincial level in Vietnam. Our research, therefore, was conducted to determine the characteristics of HIV/AIDS patients, assess the therapy adherence rate and investigate the factors associated with ART adherence of HIV/AIDS patients in a provincial hospital in Vietnam in 2019.

## 2. Materials and Methods

Our cross-sectional study was conducted on all patients having HIV/AIDS in an outpatient clinic of a provincial hospital in Vietnam from June to December 2019. Eligible patients had confirmed HIV-positive results and were aware of their HIV status. By using a systematic random sampling method, participants were selected from a patient list in numerical order. The sample size was 334 patients, which was calculated using the formula for a single proportion with the estimated HIV/AIDS treatment adherence rate in Vietnam, which was 0.68, type I error probability of 5% and a confidence level of 95%. An additional 5% were elected to tolerate some participants who were lost to follow-up or discontinued treatment; thus, there were 350 patients in this study.

Patients who were 18 years of age or older were included in the study if they had undergone (ART) for at least 6 months. Additionally, the outpatient department-treated cases with complete medical records and HIV-positive test results were also included. We excluded patients with (1) mental health issues that affect their abilities to respond, (2) follow-up incapability or (3) that were deceased at the time of the study. Any patient who did not agree to sign the patient’s consent was also excluded.

The data collection began with screening and filtering medical records of patients (≥18 years old) who had at least a 6-month follow-up. A list of 802 eligible patients was formed and every 2nd person on that list would be chosen as a participant. After systematic random sampling from the list, patients’ information was extracted and filled in a specific research form with an ID. In the next step, the patients who came to the outpatient clinic at the time of research and had medical IDs coinciding with those in our form were invited to an interview. A pilot survey was performed on 30 participants who were not included in the sample to verify the appropriateness and efficacy of the proposed data collecting methods. Data collection, coding and patient interviews were conducted and strictly controlled by the research team to ensure data consistency.

Patient adherence was measured and assessed by using a questionnaire which was taken as reference from the AIDS Clinical Trials Group (ACTG) Adherence and WebAd-Q Questionnaire [7,8]. In addition to the patient’s general information, the queries focused on their (1) adherence behaviors, (2) current medical status, (3) awareness of HIV care and treatment and (4) the support from medical services.

Patients were considered as having good knowledge of ART if they obtained 8 points (1 point for each correct answer) in the “treatment adherence knowledge” section from the questionnaire [9]. Queries in this section were about whether patients knew (1) what ARV (antiretroviral) drugs were, (2) how long it took for ARV treatment, (3) how many types of drugs that ARV was combined with, (4) how to take ARV drugs, (5) the side effects of drugs, (6) how to deal with the side effects, (7) how to deal with missing doses and (8) how to calculate the time to take the missing dose after remembering.

The adherence rate was determined as the number of adherent patients divided by the total number of patients at the time of the study. Patients were assessed as “adherent” when they fulfilled all the following criteria: (1) take drugs correctly with appropriate dose as prescribed, (2) on time and (3) suitably with food and drink, and (4) adherent with clinic visits [10]. Patients were considered non-adherent when they did not meet at least one of those requirements. In terms of factors associated with patients’ non-adherence, prior studies indicated that the most common causes were alcohol consumption, drug and substance abuse, working away from home and lack of good knowledge about ART adherence and family support [11,12,13]. Thus, in addition to patients’ knowledge and adherence, our questionnaire also considered the aforementioned factors.

Descriptive statistics were used to summarize categorical (proportions and frequencies) and continuous variables (means, max, min and standard deviations). The analysis results were presented in the form of a corresponding table. To analyze data and assess the difference between groups, the chi-square test with odds ratios (OR) and 95% confidence intervals (CIs) was performed. Univariable analysis results which were *p*-values ≤ 0.1 were analyzed by multivariable logistic regression with the Forward likelihood ratio (LR) method and also by the Backward LR method with the comparison standard based on the likelihood ratio to confirm the data accuracy. The goodness of fit for logistic regression models was evaluated by the Hosmer–Lemeshow test. Data were entered using Microsoft Excel and results were processed using SPSS version 20.0 (IBM Corp., New York, the United States). The outcomes were statistically significant if the *p*-values were ≤ 0.05.

Ethical approval for this study was obtained from the Medical Ethics Councils of Can Tho University of Medicine and Pharmacy and the provincial hospital (Approval number: 21/HDDD) in February 2019. Patients were clearly informed of the purpose and procedure of the study and voluntarily signed an informed consent form. Personal information of participants was kept strictly confidential in accordance with The Human Immunodeficiency Virus and Acquired Immune Deficiency Syndrome (Prevention and Control) Act.

## 3. Results

**Figure 1:**
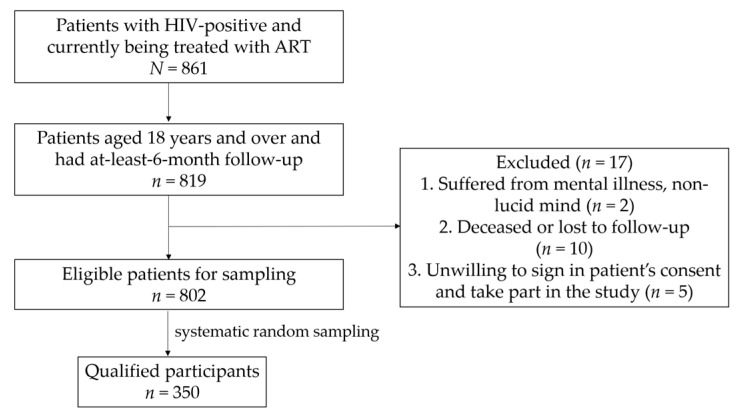
Diagram of the research steps.

The number of patients with HIV who were being treated in the hospital was 861, of whom 95% were 18 years of age or older and had at least 6 months of follow-up on ART (Figure 1).

From the aforementioned group, 17 patients were excluded due to the exclusion criteria. After systematic random sampling from the list and selecting an additional 5%, 350 patients were included for this study.

### 3.1. Demographic and ART-Related Characteristics of Patients with HIV 

In our study, the majority of patients were male (62.9%), and the dominant age group (≥35 years old) accounted for 53.7% (Table 1). In total, 47.4% of participants were married while the rest were single, divorced, separated or widowed. The number of patients who lived far away from the clinic (home-to-clinic distance ≥ 20 km) and worked far from their homes was 242 (69.1%) and 135 (38.6%), respectively.

Sexual intercourse was the primary route of transmission of HIV (95.1%) (Table 1). Only 10 (2.9%) patients were at risk for opportunistic infection whereas a much larger proportion (89.1%) of HIV participants were co-infected with both hepatitis B virus (HBV) and hepatitis C virus (HCV) previously. The proportion of HIV patients that had a history of drug abuse accounted for 30.3%.

The rate of medication adherence increased with age; patients who were over 35 years old had better compliance than those who were younger (*p* = 0.03) (Table 1). Furthermore, there was a significant correlation between marital status and high adherence in patients, particularly for those who were married (*p* = 0.001). Intriguingly, patients who had not partaken in substance abuse were more likely to be well-adherent than those who were addicted (*p* = 0.001). However, there was no significant difference between the treatment adherence and side effects of medicines in patients, the period of treatment or with the gender of participants (*p* > 0.05).

### 3.2. The Proportion of Adherence to ART

The majority of patients were strictly adherent to their prescription (Table 2). The proportions of participants who took the right medicine and the appropriate dose were 98.3% and 86.3%, respectively. Furthermore, 87.4% of patients took ARV drugs on time, and 94.9% of participants took medicine appropriately in combination with food and beverage. The percentage of patients who kept up with their medical appointments was 99.4%. Notably, 75.7% of the patients had high adherence to ART (fulfilled all four criteria).

### 3.3. Determinants of Patients’ Adherence

The multivariable logistic regression was analyzed with 13 factors of which simple regression analysis results were *p*-values ≤ 0.1. The factors that showed significant differences (*p*-value ≤ 0.05) between patients’ features and ART adherence were as follows: marital status, working away from home, substance use status, general knowledge about ART treatment, stopping use of ARV drugs after improvement and self-assessment of adherence with therapy (Table 3).

A multivariate logistic regression analysis was used on 13 variables: (1) age, (2) marital status, (3) work away from home, (4) opportunistic infection, (5) drug abuse, (6) general knowledge about ART treatment, (7) patients with stress, anxiety or depression, (8) patients’ quality of life after treatment, (9) patients wanting to stop using drugs after improvement, (10) prompting of medication, (11) waiting time for medical examination, (12) satisfaction with medical services and (13) self-assessment of adherence to treatment by the Forward LR method.

## 4. Discussion

In our study, the adherence rate of participants was 75.7%. The factors marital status, being away from home, substance abuse, general knowledge about ART, stopping medication after improvement and self-assessment of therapy adherence were significantly associated with adherence in patients with HIV.

Adherence to therapy was considered the most pivotal element which could greatly affect patients’ health outcomes. Our study calculated that the adherence rate of participants with HIV was 75.7% (Table 2). This finding is similar to the studies carried out in other provinces with reported adherence rates of 71.8% and 77% [14,15]. Yet, two prior cross-sectional studies in Vietnam showed reported adherences of 62.9% and 68.4%, which were lower than our findings [16,17]. The reasons for the discrepancy in the adherence rates might be the sample sizes of their studies, which were smaller than ours.

In a detailed look, the proportion of patients who did not take enough dosage accounted for 13.7%, which was slightly higher than that of other research (11.3% and 9.5%) [16,17]. Yet, our results showed a lower adherence rate in terms of taking drugs on time (12.6%) than that of those two studies (22.7% and 28.5%). The measurement methods and the sampling intervals of the questionnaire may cause these differences.

Our multivariable regression analysis indicated that married patients were more strictly adherent to therapy than those who were single (*p* < 0.001 and *p* = 0.007 in univariable and multivariable regression analyses, respectively) (Table 3). Patients with HIV were supported not only in psychological well-being and financial status but also with medical care and daily activities. The significant correlation between partner support and adherence to ART was well documented; women were more likely to have low adherence if they had an unsupportive male partner [18,19].

Our research also attributed poor adherence to working away from home (*p* = 0.011). Various work-related reasons that lead to non-adherence were revealed [13]. Particularly, the most common causes were due to an intense schedule and fear of stigma at the patients’ workplaces. Moreover, internalized HIV-related stigma was proven to result in sub-optimal ART adherence [20]. Thus, individuals who worked away from their homes tended to be poorly adherent to treatment in comparison with those who did not.

Additionally, the recent use of alcohol or other stimulants has been a major barrier to ART adherence. Our findings were consistent with other research in the United States, Africa and Uganda [21,22,23,24]. The non-adherence in men with frequent use of alcohol was due to cognitive impairment and the intentional skipping of medication when drinking [25]. To sum up, having a comprehensive understanding of patients’ behaviors which lead to their poor adherence could be informative in intervening treatment and medication scheduling.

The direct interview method in our research minimized the ability of question misunderstanding and data replication. Besides, our investigation took reference from established and prevalent questionnaires which were considered to be effective in the assessment. In addition, the relatively large sample size allowed us to have statistical efficiency in examining ART-related factors in a multivariable regression analysis. Yet, the data might not be generalized at the national level since we conducted our investigation on one provincial hospital. Additionally, selection bias may have occurred in our study due to the exclusion of lost-to-follow-up patients with HIV and non-participating patients. These non-participants may require special attention after this study; a follow-up study will focus on psychological barriers and stigmatization of this group of patients.

The identification of factors associated with non-adherence in patients plays a critical role in addressing ART-related issues. Our research showed that various reasons contribute to non-adherence, e.g., drug abuse, being away from home and medication’s adverse effects. Thus, understanding those barriers could have major benefits for both the patients and healthcare providers in strengthening HIV care and treatment strategies. Further research should consider more methods and approaches as well as strategies of management to improve treatment adherence in HIV patients.

## 5. Conclusions

Three-quarters of patients were adherent to ART in southern Vietnam. Various factors were demonstrated to be correlated with patients’ adherence. Researchers should consider these determinants of adherence in developing interventions in further studies to improve treatment outcomes.

## 6. Patents

This section is not mandatory but may be added if there are patents resulting from the work reported in this manuscript.

## Figures and Tables

**Table 1:** Study population characteristics.

Characteristics	Frequency and Percentage (n, %)	Adherence		*p*-Value **
		Yes (n, %)	No (n, %)	
**Age**				
≥35 years old	188 (53.7)	151 (80.3)	37 (19.7)	0.03
<35 years old	162 (46.3)	114 (70.4)	48 (29.6)	
**Gender**				
Male	220 (62.9)	165 (75.0)	55 (25.0)	0.685
Female	130 (37.1)	100 (76.9)	30 (23.1)	
**Marital status**				
Married	166 (47.4)	140 (84.3)	26 (15.7)	<0.001
Single	184 (52.6)	125 (67.9)	59 (32.1)	
**Currently living with**				
Relatives, friends	295 (84.3)	225 (76.3)	70 (23.7)	0.574
Alone	55 (15.7)	40 (72.7)	15 (27.3)	
**Home-to-clinic distance**				
<20 km	108 (30.9)	74 (68.5)	34 (31.5)	0.036
≥20 km	242 (69.1)	191 (78.9)	51 (21.1)	
**Level of education**				
Upper secondary	206 (58.9)	161 (78.2)	45 (21.8)	0.203
Lower secondary	144 (41.1)	104 (72.2)	40 (27.8)	
**Occupation**				
Employment	285 (81.4)	216 (75.8)	69 (24.2)	0.945
Unemployment	65 (18.6)	49 (75.4)	16 (24.6)	
**Work away from home**				
Yes	135 (38.6)	94 (69.6)	41 (30.4)	0.035
No	215 (61.4)	171 (79.5)	44 (20.5)	
**HIV transmission route**				
Sexual behaviors	333 (95.1)	255 (76.6)	78 (23.4)	0.142 *
Drug abuse and mother–child	17 (4.9)	10 (58.8)	7 (41.2)	
**Clinical stage during treatment**				
Stage I, II	341 (97.4)	257 (75.4)	84 (24.6)	0.693 *
Stage III, IV	9 (2.6)	8 (88.9)	1 (11.1)	
**CD4 count (cells/mm^3^)**				
≥200	283 (83.7)	212 (74.9)	71 (25.1)	0.272
<200	55 (16.3)	45 (81.8)	10 (18.2)	
**Opportunistic infection**				
Yes	10 (2.9)	5 (50.0)	5 (50.0)	0.067 *
No	340 (97.1)	260 (76.5)	80 (23.5)	
**Hepatitis B and C virus coinfection**				
Yes	312 (89.1)	236 (75.6)	76 (24.4)	0.927
No	38 (10.9)	29 (76.3)	9 (23.7)	
**ART regimen**				
First-line ART	340 (97.1)	256 (75.3)	84 (24.7)	0.461 *
Second-line ART	10 (2.9)	9 (90.0)	1 (10.0)	
**Viral load (copies/mL)**				
<20 cps	305 (89.7)	230 (75.4)	75 (24.6)	0.821
>20 cps	35 (10.3)	27 (77.1)	8 (22.9)	
**Dosing times**				
Once a day	299 (85.4)	224 (74.9)	75 (25.1)	0.399
Twice a day	51 (14.6)	41 (80.4)	10 (19.6)	
**Drug abuse**				
Yes	244 (69.7)	173 (70.9)	71 (29.1)	0.001
No	106 (30.3)	92 (86.8)	14 (13.2)	
**Drug side effects**				
Yes	304 (86.9)	228 (75.0)	76 (25.0)	0.423
No	46 (13.1)	37 (80.4)	9 (19.6)	
**General knowledge about ART**				
Yes	217 (62.0)	180 (82.9)	37 (17.1)	<0.001
No	133 (38.0)	85 (63.9)	48 (36.1)	
**Patients with stress, anxiety, depression**				
Yes	160 (45.7)	128 (80.0)	32 (20.0)	0.086
No	190 (54.3)	137 (72.1)	53 (27.9)	
**Patients’ quality of life after treatment**				
Better	239 (68.3)	197 (82.4)	42 (17.6)	<0.001
Normal	111 (31.7)	68 (61.3)	43 (38.7)	
**Patients want to stop using drugs after improvement**				
Yes	56 (16.0)	28 (50.0)	28 (50.0)	<0.001
No	294 (84.0)	237 (80.6)	57 (19.4)	
**Prompting of medication**				
Self-managing	293 (83.7)	229 (78.2)	64 (21.8)	0.016
Spouses, parents, siblings	57 (16.3)	36 (63.2)	21 (36.8)	
**Waiting time for medical examination**				
Fast	143 (40.9)	120 (83.9)	23 (16.1)	0.003
Normal	207 (59.1)	145 (70.0)	62 (30.0)	
**Satisfaction of medical services**				
Very satisfied	188 (53.7)	160 (85.1)	28 (14.9)	<0.001
Satisfied	162 (46.3)	105 (64.8)	57 (35.2)	
**Feeling uncomfortable with treatment adherence**				
Yes	37 (10.6)	27 (73.0)	10 (27.0)	0.681
No	313 (89.4)	238 (76.0)	75 (24.0)	
**Self-assessment of adherence to treatment**				
High adherence	282 (80.6)	240 (85.1)	42 (14.9)	<0.001
Medium and low adherence	68 (19.4)	25 (36.8)	43 (63.2)	
**Treatment period (months)**				
Months (mean ± SD)	350 (100)	48.89 ± 36.13	51.25 ± 33.22	0.593 ***

The chi-square test was performed to determine the relationship between adherence and related factors. * Fisher’s Exact Test. ** *p*-value ≤ 0.05 was considered statistically significant. *** Univariable regression logistic.

**Table 2:** Adherence to antiretroviral therapy (ART) among patients with HIV.

Adherence Assessment	Frequency (*n* = 350)	Percentage (%)
**Take the right medicine**		
Yes	344	98.3
No	6	1.7
**Take correct dose**		
Yes	302	86.3
No	48	13.7
**Take medicine on time**		
Yes	306	87.4
No	44	12.6
**Take medicine properly in combination with eating and drinking**		
Yes	332	94.9
No	18	5.1
**Routine follow-up**		
Yes	348	99.4
No	2	0.6
**Adherence to treatment**		
Yes	265	75.7
No	85	24.3

**Table 3:** Univariable and multivariable regression analyses of the correlation between patients’ characteristics and ART adherence.

Features	Univariable Regression Logistic		Multivariable Regression Logistic	
	OR (95%CI)	*p*-Value	OR (95%CI)	*p*-Value
**Marital Status**				
Married	2.54 (1.51–4.28)	<0.001	2.29 (1.25–4.19)	0.007
Single *	-	-	-	-
**Work away from home**				
No	1.7 (1.03–2.78)	0.036	2.15 (1.19–3.89)	0.011
Yes *	-	-	-	-
**Drug abuse**				
No	2.7 (1.44–5.05)	0.002	2.03 (1.01–4.08)	0.048
Yes *	-	-	-	-
**General knowledge about ART treatment**				
Yes	2.75 (1.67–4.53)	<0.001	1.99 (1.1–3.64)	0.026
No *	-	-	-	-
**Stop using drugs after improvement**				
No	4.16 (2.29–7.56)	<0.001	2.16 (1.04–4.46)	0.039
Yes *	-	-	-	-
**Self-assessment of adherence to treatment**				
High adherence	9.83 (5.44–17.77)	<0.001	5.97 (3.07–11.61)	<0.001
Medium and low adherence *	-	-	-	-

(*): reference group.

## Data Availability

Data sharing is not applicable.
